# The identification of informative genes from multiple datasets with increasing complexity

**DOI:** 10.1186/1471-2105-11-32

**Published:** 2010-01-15

**Authors:** S Yahya Anvar, Peter AC 't Hoen, Allan Tucker

**Affiliations:** 1 Center for Intelligent Data Analysis, School of Information Systems, Computing and Mathematics, Brunel University, Uxbridge, Middlesex, UB8 3PH, UK; 2 Center for Human and Clinical Genetics, Leiden University Medical Center, P.O. Box 9600, 2300 RC Leiden, The Netherlands

## Abstract

**Background:**

In microarray data analysis, factors such as data quality, biological variation, and the increasingly multi-layered nature of more complex biological systems complicates the modelling of regulatory networks that can represent and capture the interactions among genes. We believe that the use of multiple datasets derived from related biological systems leads to more robust models. Therefore, we developed a novel framework for modelling regulatory networks that involves training and evaluation on independent datasets. Our approach includes the following steps: (1) ordering the datasets based on their level of noise and informativeness; (2) selection of a Bayesian classifier with an appropriate level of complexity by evaluation of predictive performance on independent data sets; (3) comparing the different gene selections and the influence of increasing the model complexity; (4) functional analysis of the informative genes.

**Results:**

In this paper, we identify the most appropriate model complexity using cross-validation and independent test set validation for predicting gene expression in three published datasets related to myogenesis and muscle differentiation. Furthermore, we demonstrate that models trained on simpler datasets can be used to identify interactions among genes and select the most informative. We also show that these models can explain the myogenesis-related genes (genes of interest) significantly better than others (*P *< 0.004) since the improvement in their rankings is much more pronounced. Finally, after further evaluating our results on synthetic datasets, we show that our approach outperforms a concordance method by Lai *et al*. in identifying informative genes from multiple datasets with increasing complexity whilst additionally modelling the interaction between genes.

**Conclusions:**

We show that Bayesian networks derived from simpler controlled systems have better performance than those trained on datasets from more complex biological systems. Further, we present that highly predictive and consistent genes, from the pool of differentially expressed genes, across independent datasets are more likely to be fundamentally involved in the biological process under study. We conclude that networks trained on simpler controlled systems, such as *in vitro *experiments, can be used to model and capture interactions among genes in more complex datasets, such as *in vivo *experiments, where these interactions would otherwise be concealed by a multitude of other ongoing events.

## Background

High-throughput gene expression profiling experiments have increased our understanding of the regulation of biological processes at the transcriptional level. In bacteria [1] and lower eukaryotes, such as yeast [2], modeling of regulatory interactions between large numbers of proteins in the form of regulatory networks has been successful. A regulatory network represents relationships between genes and describes how the expression level, or activity, of genes can affect the expression of other genes. The network includes causal relationships where the protein product of a gene (e.g. transcription factor) directly regulates the expression of a gene but also more indirect relationships. Modeling has been less successful for more complex biological systems such as mammalian tissues, where models of regulatory networks usually contain many spurious correlations. This is partly attributable to the increasingly multi-layered nature of transcriptional control in higher eukaryotes, e.g. involving epigenetic mechanisms and non-coding RNAs. However, a potential major reason for the decreased performance is due to *biological complexity *of datasets which can be defined as the increase of biological variation and the presence of different cell types, which is not compensated by an increase in the number of replicate data points available for modeling. There is an urgent need to identify regulatory mechanisms with more confidence to avoid wasting laborious and expensive wet-lab follow-up experiments on false positive predictions.

The main paradigms of this paper are that regulatory interactions that are consistently found across multiple datasets are more likely to be fundamentally involved and that these regulatory interactions are easier to find in datasets with less biological variation. In the end, regulatory networks trained on less complex biological systems could thus be used for the modeling of the more complex biological systems. We do this using a novel computational technique that combines Bayesian network learning with independent test set validation (using error and variance measures) and a ranking statistic. Whilst Bayesian networks and Bayesian classifiers have been used with great success in bioinformatics [3,4], an important weakness has been that, when trying to build models that reveal genuine underlying biological processes, a highly accurate *predictive *model is not always enough [5]. The ability to *generalize *to other datasets is of greater importance [6]. Simple cross-validation approaches on a single dataset will not necessarily result in a model that reflects the underlying biology and therefore will not generalize well. Our approach is to exploit multiple datasets of increasingly complex systems in order to identify more informative genes reflecting the underlying biology.

Bayesian networks have been an important concept for modeling uncertain systems [7-10]. In the last decade several researchers have examined methods for modeling gene expression datasets based on Bayesian network methodology [2-4]. These networks are directed acyclic graphs (DAG) that represent the joint probability distribution of variables efficiently and effectively [11]. Each node in the graph represents a gene, and the edges represent conditional independencies between genes. Bayesian networks are popular tools for modeling gene expression data as their structure and parameters can easily be interpreted by biologists.

Bayesian classifiers are a family of Bayesian networks that are specifically aimed to classify cases within a data set through the use of a class node. The simplest is known as the naïve Bayes classifier (NBC) where the distribution for every variable is conditioned upon the class and assumes independence between the variables. Despite this oversimplification, NBCs have been shown to perform very competitively on gene expression data in classification and feature selection problems [5,12,13]. Other Bayesian classifiers, which often have higher *model complexity *as they contain more parameters, involve learning different networks such as trees between the variables and therefore relax the independence assumption [11]. The logical conclusion is the general Bayesian Network Classifier (BNC) which simply learns a structure over the variables including the class node. In this paper, we explore the use of the NBC, and the BNC for predicting expression on independent datasets in order to identify informative genes using classifiers of differing complexity.

Accordingly, in order to optimize the classifier and choose the best method, we need to consider the classifiers' bias and variance. Since bias and variance have an inverse relationship [12], which means decreasing in one increases the other, cross-validation methods can be adopted in order to minimize such an effect. The k-fold cross-validation [12,14] randomly splits data into k folds of the same size. A process is repeated k times where k-1 folds are used for training and the remaining fold is used for testing the classifier. This process leads to a better classification with lower bias and variance [15] than other training and testing methods when using a single dataset. In this paper, we exploit bias and variance using both cross-validation on a single dataset and also independent test data in order to learn models that better represent the true underlying biology. In the next section we provide a description of the gene identification algorithm for identifying gene subsets that are specific to a single simple dataset as well as subsets that exist across datasets of all biological complexity. We used den Bulcke *et al*. [16] proposed model for generating synthetic datasets to validate our findings on real microarray data. Moreover, we evaluate the performance of our algorithm by comparing the ability of this model in identifying the informative genes and underlying interactions among genes with the concordance model. Finally, we present the conclusion and summary of our findings in the last section.

## Methods

### Multi-Data Gene Identification Algorithm

The algorithm involves taking multiple datasets of increasing biological complexity as input and a repeated training and testing regime. Firstly, this involves a k-fold cross-validation approach on the single simple dataset (from now on we refer to this as the cross-validation data) where Bayesian networks are learnt from the training set and tested on the test set for all k folds. These folding arrangements have been used again for assessing a final model. The Bayesian Network learning algorithm is outlined in the next section.

The Sum Squared Error (SSE) and variance is calculated for all genes over these folds by predicting the measured expression levels of a gene given the measurements taken from others. Next, the same models from each k fold are tested on the other (more complex) datasets (the independent test data) and SSE and variance are again calculated. These SSE and variances are used to rank the genes according to their informativeness (which represents the most predictive and influential genes). Those that are ranked highly in the single-dataset cross-validation experiments will be informative, specific to the single datasets experiment, whereas those that are ranked highly on the independent datasets should be informative in a more general sense in that they are predictive (low SSE) and consistent (low variance) across datasets of all complexity. We evaluate the statistical significance of these rankings using a method proposed by Zhang *et al*. [17]. The full details are outlined in Algorithm 1 where *TrainD *represents the training data (cross-validation data, here the relatively simple datasets), and *TestD_1 _... TestD_M _*represent the more complex test datasets, independent test data.

### Bayesian Network Structure Learning

The goal of learning gene regulatory networks using Bayesian network approaches is to establish the structure of the network and then to parameterize the conditional probability tables [18]. As the number of possible network structures is huge, learning the structure of a network has a high computational cost. Since the effective learning of network structure engages a trade-off of bias vs. variance, the necessity of designing an algorithm in which it can generate an ideal structure for a given dataset, with a degree of biological complexity, is crucial [19]. In this study, instead of using well studied but unrealistic and sometimes not effective classifiers such as NBC and Tree Augmented Networks (TAN), we use an optimization approach that uses a simulated annealing search and the Bayes Information Criterion (BIC) as a scoring metric [20]. The advantage of simulated annealing over other methods (like greedy searches or hill climbing) is that it aims to avoid local maxima [11]. We have chosen the BIC as a fitness function as it is less prone to overfitting through the use of a penalizing term for overly complex models.

Bayesian networks with more connections between their nodes require a higher number of parameters and as a result increase the complexity of the models exponentially [21]. Therefore, we explore three different classes of model learning: the Selective Naïve Bayes (SNB) where only links between a class node representing differentiation status and a gene are explored, a search that explores structures with links between genes but limiting each gene to having only one parent (1PB). Limiting the number of parents in a Bayesian network is common practise but can be considered a crude approach to reducing parameters. As a result we also explore a full unlimited structure learning (NPB) and learn these structures using the simulated annealing with the BIC scoring metric (which naturally penalises overly complex networks). In this study, the initial state of the structure is an empty DAG with no link. In order to alter the network structures, three operators have been used within the simulated annealing. These operators are adding, removing, or swapping links to generate a new network for validation. These alterations can be either accepted or rejected. The outline of this procedure can be found in Algorithm 2.

### Prediction and Ranking

Zhang *et al*. [17] proposed a method to convert a set of gene rankings into position p-values to evaluate the significance of a given gene. However, this involved working with resampling techniques upon a single dataset. Here, we use the ranking lists according to the model's average SSE and variance for both the original simple dataset and the independent test sets in order to generate position p-values. This requires us to include, a number of random genes which can be counted as uninformative genes. By comparing the actual ranking of the gene with the null distribution we can calculate the position p-values. In this paper we are using three independent datasets so we do not need to use resampling in order to generate more gene rankings as Zhang *et al*. [17] did in their experiments. In addition, the different rankings will have different interpretations as some are based purely on the simple dataset whilst others are influenced by error and variance on the more biologically complex independent data.

### Datasets

With the aim of investigating the influence of the complexity of a gene expression dataset on the performance of classifiers in identifying the gene regulatory network, three gene expression datasets (with increasing biological variation) have been chosen for this study (GSE3858 [22], GSE1984 [23], and GSE989 [24]). These three datasets are all concerned with the differentiation of cells into the muscle (Myogenic) lineage. During this process, mononucleated precursor cells stop to proliferate, differentiate and fuse with each other to become elongated multinucleated myotubes or myofibres. This in-vitro system mimics the formation of new muscle fibres in-vivo. The cell types differ between the different datasets:

• GSE3858: Embryonic fibroblasts (EF)

• GSE989 and GSE1984: C2C12 tumor cell line that has the potential for differentiation into different mesodermic lineages (mainly muscle and bone)

Also methods to drive cells into myogenic differentiation differ:

• GSE3858: Exogenous expression of the myogenic transcription factors are Myod and Myog.

• GSE989 and GSE1984: Serum Starvation

In addition, the study by Sartorelli included different treatments that affect the timing and efficiency of the myogenic differentiation process. The time points for sampling differ between the studies (Table 1). The class node reflecting the differentiation status had two possible states: undifferentiated (for all time points until myogenic differentiation was induced) and differentiated (for time points where myogenic differentiation had been induced). In the rest of this paper we call these datasets by the name of the first author (e.g. Cao instead of GSE3858).

**Table 1:** Specification of three muscle differentiation datasets

Dataset	Cell Type	Platform	Samples	Time Points
Tomczak	C2C12	Affy U74A	24	8
Cao	EF	Affy 430.2	36	4
Sartorelli	C2C12	Affy U74A	32	6

### Data Processing and Analysis

The raw microarray data were normalized and summarized with the RMA method [25], using the affy package in R. Only the 8904 probesets common to the Affymetrix U74A and 430.2 used in mentioned studies were considered in the analysis. All datasets were standardized to mean 0 and the standard deviation 1 across the genes. For the scope of this paper, first, we selected for each dataset a subset of 100 genes most affected by the induction of differentiation. These genes were identified with Student's t-test which compared samples from undifferentiated and differentiated cell cultures, disregarding the time of differentiation. An additional 50 genes were randomly selected to be able to calculate ranking p-scores described above and using the Kolmogorov-Smirnov test. For cross-validation we divided Cao dataset into 9 folds, Sartorelli into 8 folds, and Tomczak into 6 folds based upon the number of samples in each dataset. Simulated annealing has three attributes which should be set before starting the learning phase. It is crucial to set an appropriate initial temperature, sufficient number of iterations, and a convenient fitness function. In this study, the initial temperature has been set to 10 and it terminates at 0.001. The number of iterations has been set to 1000 for the first set of experiments only using most informative genes (top 100) and then we set the number of iterations to 1500 since we added 50 uninformative genes to the network. The code is implemented in Matlab 2007a using the Bayes Net toolbox [26] to generate gene regulatory networks.

### Analysis of myogenesis-Related genes

Myogenesis-related genes are defined as genes associated with the Gene Ontology term "Muscle Development" supplemented with all genes strongly associated with Myogenesis in the biomedical literature, as determined with the literature analysis tool Anni v2.0 [27] with the association score greater than 0.02.

### Analysis of Synthetic datasets

The use of datasets in which the underlying network is known enables us to validate the new algorithms that have been developed to identify gene regulatory networks and capture the most informative genes. den Bulcke *et al*. [16] proposed a new methodology to generate synthetic datasets where the network structure is known and biological, experimental, and model complexity can be manipulated. However, a disadvantage of this approach is that the generated networks can contain some overlapping pieces of the known network which may weaken the models being probabilistically independent [28]. Whilst SynTReN uses resampling from potentially overlapping networks, the generated data undergoes a robust statistical cross-validation regime ensuring that any prediction is applied to unseen data. The focus of this paper is upon the prediction of increasingly complex datasets, sampled from some underlying biological process. Consequently, these synthetic datasets can be used for validating the performance of our methodology in identifying the informative genes and the interactions among them in real microarray data. SynTReN [16] generates networks with more realistic topological characteristics and since we use this application to investigate the impacts of biological, experimental, and model complexity on identifying informative genes using the same sub-network is an advantage. Three datasets have been generated on the well-described network structure of *E. coli *[29] which contains 1330 number of nodes and 2724 interactions. These datasets have been generated in a manner that they can match the key characteristics of real microarray datasets we used in this study (for instance, limiting the number of genes that were selected for modelling to 150). This enables us to investigate the possibility of reproducing similar results on synthetic data which can be easily corrected for differences such as number of samples and time points per dataset (see Additional file 1) and avoid weakening the probabilistically independent assumption of the generated datasets.

### Analysis of Concordance between datasets

The study of the concordance between microarray datasets has increased considerably in the past few years [30]. However, a robust statistical method for examining the concordance or discordance among microarray experiments carried out in different laboratories is yet to develop. Methods such as multiplication of gene p-values in order to generate a list of rankings for concordance genes showed bias towards datasets with higher significance level [31]. Lai *et al*. [32] proposed a promising methodology (which we call concordance model) to investigate the concordance or discordance between two large-scale datasets with two responses. This method uses a list of z-scores, generated using a statistical test of differential expression, as an input to evaluate the concordance or discordance of two datasets by calculating the mixture model based likelihoods and testing the partial discordance against concordance or discordance. Additionally, the statistical significance of a test is being evaluated by the parametric bootstrap procedure and a list of gene rankings is being generated which can be used for integrating two datasets efficiently. In this paper we are using a set of gene rankings generated by this method to evaluate the performance of our model in identifying informative genes from multiple datasets with increasing complexity.

## Results

The aim of this study is to demonstrate firstly, the influence of model complexity in discovering accurate gene regulatory networks on multiple datasets with increasing biological complexity. Secondly, to investigate if cleaner and more informative datasets can be used for modelling more complex ones. Therefore, three public datasets that are concerned with the differentiation of cells into muscle lineage were chosen for this study. From a biological point of view, Sartorelli is the most complex dataset since it involves different treatments influencing myogenesis. Tomczak and Cao are less complex datasets. It is difficult to say how their complexity relates since Tomczak uses more heterogeneous stimuli to induce differentiation but has more time points, while Cao uses more defined stimuli (Myod or Myog transduction) and less time points. In order to meet the scope of this study, we evaluated the quality and informativeness of these datasets based on two criteria. Firstly, we calculated the average correlations between replicates as a measurement of noisiness of each dataset. Secondly, using Student's t-test method, we counted the number of differentially expressed genes with the significance levels of 0.05 and 0.01 as a measurement of informativeness (Table 2). Although the average correlations between replicates in all three datasets are very close, datasets differ in number of significant genes they hold. Tomczak is the most informative dataset as it includes the most number of significant genes and has a higher average correlation value for the replicate samples in the dataset which represent the lowest level of noise. In contrast, Sartorelli contains the least differentially expressed genes with almost 12% of what Tomczak contains. Moreover, it has the lowest average correlation value and can be marked as the most complex dataset to model in this study as it has the highest noise level and the least number of informative genes. Therefore, we ordered these datasets by increasing biological complexity in the following way: Tomczak, Cao, and Sartorelli.

**Table 2:** The average correlations between replicates and number of differentially expressed genes (based on BH corrected p-values) in each dataset

		Genes with a P-value (BH) less than	
Dataset	Correlation	0.05	0.01
Tomczak	0.975	4602	3604
Cao	0.971	3668	2623
Sartorelli	0.964	1199	458

### Comparison of classifiers and network analysis

**Figure 1:**
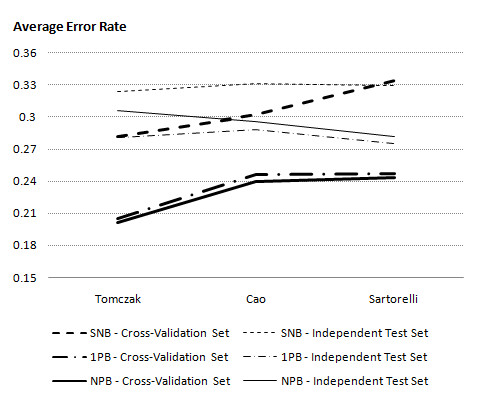
**The comparison of classifiers with increasing model complexity**. Three Bayesian network models (SNB, 1PB, and NPB) have been trained using cross-validation set and validated on independent datasets. An average error rate of the classifiers' prediction has been calculated for each gene and an overall SSE on cross-validation set and independent test set are illustrated in this figure.

We now explore how the different classifiers performed on these three datasets. Figure 1 shows the average error rate of the different classifiers trained on each given dataset. It can be seen that of the three classifiers, 1PB and NPB generated the same pattern and have very close error rates on cross-validation (training) sets. However, it is evident that NPB (particularly on Tomczak) performs poorer than 1PB on the independent test set, possibly due to overfitting as these models contain more parameters. Even though SNB performed poorly on both the cross-validation test and the independent data test, in some cases it could compete with NPB which appears to be too complex to predict some of the independent datasets accurately. Hence, 1PB has performed favorably, both in terms of average error rate and the difference between the cross-validation test and the independent data test (see Additional file 1 for complete set of results).

According to Mac Nally [33] simple models should be sought for various reasons. Firstly, simple models are more stable and capable of not overfitting to noise in the data which will influence the performance of classifier with future data. Secondly, they tend to provide a better insight into causality and interactions among genes. Finally, reducing the number of parameters will decrease the cost of validating a model for current and future data. However, we need a model that matches the complexity of data sets. Considering this argument along with our first set of results, we chose 1PB as a model that can capture the interactions among genes and does not overfit to noise. In order to understand the impacts of using different datasets for gene selection and training 1PB classifier (which will be discussed in the next section), we need to analyse the performance of the 1PB classifier on the top 100 (most informative) genes in more detail.

Additional file 1, Figure S7 represents the comparison of the error rate of the 1PB classifier on cross-validation versus the independent test. It is shown that the 1PB classifier trained on Tomczak performed significantly better on cross-validation and Sartorelli shows the lowest differentiation between cross-validation and the independent test with almost the same average error rate on the cross-validation set compared to Cao. Although the differentiation of average error rate on the cross-validation set and independent test set is high in Tomczak, this model produced the best models in terms of the lowest overall error rate. This figure raises the idea that Tomczak is the most informative dataset since it can model any dataset, regardless of the gene selection method, significantly better than the other alternatives. This will be discussed in more detail in the *Extraction of infotmative genes *section.

### Comparison of gene selections with differing informativeness

**Figure 2:**
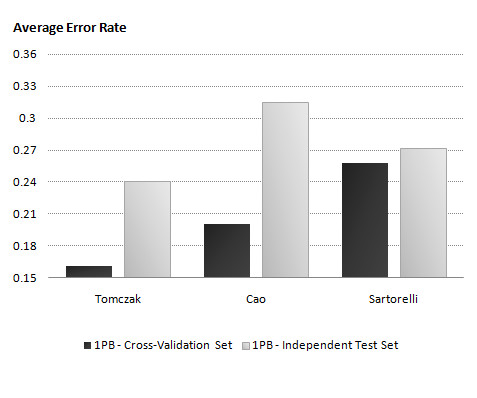
**Evaluating the accuracy of 1PB using different datasets for gene selection**. We selected genes using only one dataset (black) at a time and compared the average error rate of 1PB classifier learnt and trained on a same dataset and validated on the other two datasets independently (grey).

We now look into how the different gene selections impact on the average error rate of the 1PB classifier for both cross-validation and the independent test. Figure 2 demonstrates the performance of the 1PB classifier in modeling datasets generated using different gene selections. Clearly, unlike Sartorelli, genes selected from Tomczak and Cao show very good performances on cross-validation. However, by looking at the average error rate of 1PB on independent test sets, we can see that the models learnt on Cao over-fitted the data and performed poorly on the independent test set (with the SSE of 0.32) whereas Sartorelli shows the lowest differentiation between the two sets. Overall the Tomczak selection performed the best both on cross-validation and the independent test.

It is important to adopt a methodology that can generate an accurate gene regulatory network, moreover, it is crucial to generate a model that can capture the significant genes and distinguish informative genes from uninformative ones. For this purpose, we added 50 randomly selected genes with high p-values (which imply less relatedness to Myogenesis) from the distribution. This also has the effect that it will increase the complexity of the datasets.

**Figure 3:**
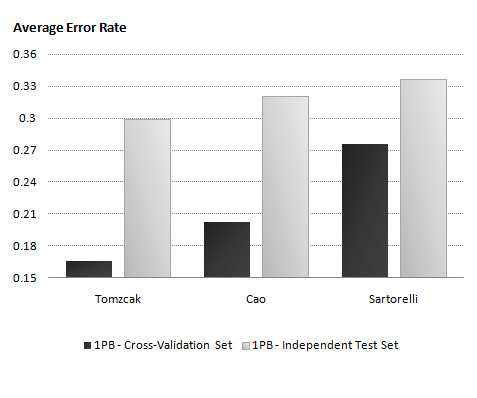
**The investigation of inference of adding more complexity to the model**. We investigated the inference of adding more complexity to the model by adding 50 randomly selected genes as uninformative on 1PB classifier performance. In this figure we compare the average error rate of 1PB classifier after adding 50 uninformative genes to the model.

Figure 3 shows that there is a similar pattern on the average error rate of cross-validation. The additional random genes do not seem to affect Cao. It does, however, have an interesting impact on Sartorelli. The models learnt on Sartorelli (see Additional file 1) performed even poorer than SNB on the independent data sets and showed no significant changes when using different datasets for training. It is interesting because we know that the Sartorelli dataset is noisy and biologically complex and adding the random genes, which increases the complexity of the models in terms of more nodes and increases the risk of spurious links, produces a classifier which appears to be unable to capture the real gene interactions. The error rate and variance of models learnt on the Sartorelli selection is significantly high in comparison with Tomczak. By comparing figures 2 and 3, we can conclude that simpler and cleaner datasets tend to perform more reliably and have more stability while increasing the complexity. Since it is important to validate these models according to their variances, we demonstrated the average variance of each model on cross-validation and the independent test set in Additional file 1, Figure S8. Interestingly, we can see a similar pattern in the classifiers' variance in comparison with the average error rate (figure 3). It is clear that we can raise the same conclusion as the simpler and cleaner datasets perform better than more noisy and complex ones. In this study, Tomczak performed favorably both in terms of bias and variance.

It is crucial to investigate if these findings are reproducible and are not prone to the number of samples and time points per dataset. Therefore, we applied our model on three synthetic datasets that have been generated by manipulating the biological, experimental, and model complexity of their known network structure using SynTReN application [16]. Additional file 1, Figure S9 illustrates that we can see a very similar pattern as we have seen on a real data where there is an increase on the average error rate of models learnt on multiple synthetic datasets with increasing biological variability. In the next section, before examining if these models can help us to capture the interactions in more complex datasets, we will investigate how well these models separate the informative genes from uninformative ones.

### Extraction of informative genes

In order to test the ability of classifiers to separate informative genes from uninformative ones, we have looked at the result of the Kolmogorov-Smirnov test (KS test) on the ranking of genes according to their average error rate using a given model. Using this algorithm, we calculated the p-value, KS test, and the result of investigating the differentiation hypothesis along with the models' bias or variance. The results of this investigation are displayed in Additional file 1, Table S1 where Cao and Tomczak performed very well on cross-validation both in terms of bias and variance. However, models learnt on Sartorelli fail to separate between informative genes and uninformative genes as the scores are generally very low.

Generally, Tomczak outperformed Sartorelli and Cao and can be chosen as the most informative dataset in this study. Models learnt on Tomczak generated the lowest bias and variance and produced the best separation. In contrast, Sartorelli is the noisiest and less informative dataset while it failed to handle any increases in complexity (both biological and model wise) and generates models with highest bias and variance which also cause disability to separate informative genes from the others. Now the question is whether we can use a simpler and cleaner dataset to model more complex ones. In the next section we show how we tackled this question.

### Analysis of the use of simpler dataset to model more complex one

**Figure 4:**
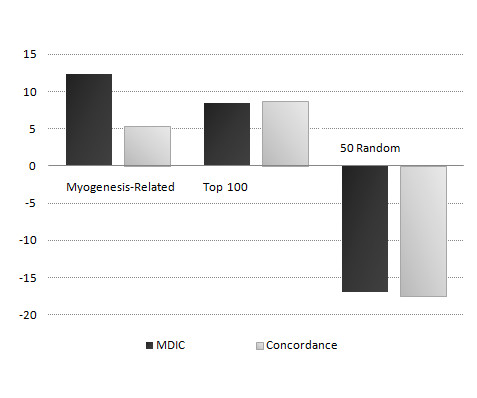
**The improvement or deterioration of genes ranking in Sartorelli**. Firstly, we selected 100 informative and 50 uninformative genes using Tomczak dataset and extracted their ranks in Sartorelli. Secondly, we trained 1PB classifier on Tomczak and tested on Sartorelli. Finally, we ranked genes according to the average error rate of 1PB classifier in predicting their values in Sartorelli. This figure illustrates the average improvement or deterioration of Myogenesis-Related, Top 100, and 50 randomly selected genes in Sartorelli generated with our method and the gene rankings generated by concordance model.

In this section, we investigate the improvement or deterioration of genes selected by Tomczak on the Sartorelli dataset. Figure 4 shows the average improvement or deterioration of ranks of myogenesis-related genes, top 100 genes (most informative), and 50 randomly selected genes (uninformative) in Sartorelli. We compared the original rank of each gene (which can be any number between 1 and 150 derived from its p-value comparing to others) with its rank based upon the ability of a model trained on Tomczak to predict gene's value in Sartorelli. Moreover, we evaluate the improvement or deterioration of genes rankings in our model with the ones generated using the concordance model described by Lai *et al*. [32]. We can clearly see that the model learnt on Tomczak can capture the informative genes in Sartorelli and improve their rank whereas uninformative genes have been pushed down (almost 17 places in average) in the ranking by the classifier. Additionally, the improvement is even more pronounced for myogenesis-related genes with 12.33 places in average, which is significantly better than others with *P *< 0.004 generated using KS test, and as expected top 100 genes has been improved by 8.44 places. Even though both methods perform similarly on improving the ranks of top 100 and deteriorating the ranks of 50 randomly selected genes, the improvement of ranks for myogenesis-related genes are much more pronounced in our model than in the concordance model (improvement of 5.38 places).

*Myh7 *and *Tor3a *are two examples of significant improvements in Sartorelli dataset. *Myh7*, which originally ranked 101, improved 96 places to rank 5 (rank 55 in concordance model). During the learning phase it has been linked to four other genes of which three of them are myogenesis-related. These genes, in both datasets, have direct correlations and can represent each other in terms of prediction and validation. However, *Tor3a *has a very low rank in both dataset and yet improved 107 places from 128 to 21 (rank 31 in concordance model). It has been linked to *Prune *which also improved 106 places (from 131 to 25, 100 in concordance model). All three genes mentioned above have been selected as informative genes from Tomczak and yet placed into the bottom 50 due to the quality of Sartorelli dataset. These were some examples of the ability of model to pull out informative genes from a distribution (figures S10a and S10b, provided in the Additional file 1).

Although the overall improvement on myogenesis-related genes is significantly high, we were concerned why this model failed to improve the rank of some genes like *Id3 *which dropped from rank 1 in Sartorelli to 133 (rank 51 in concordance model). In the learning process, *Id3 *has been linked to 4 genes which are: *Fabp3*, *Rbm38*, *X99384*, and *Slco3a1*. Now in order to answer the question, firstly, we validate the relatedness of these genes to *Id3 *in Tomczak dataset to investigate if they are significant and can represent *Id3*. Secondly, we study the expression level of these genes in Sartorelli to identify the reason why this model failed dramatically in predicting the *Id3 *value.

Additional file 1, Figure S11 demonstrates the expression level of *Id3 *along with its parent/children in both Tomczak and Sartorelli datasets. In Tomczak we can clearly see that there is an inverse relationship between *Id3 *and the other 4 genes which is very significant. While the differentiation state changes, *Id3 *drops from the expression level of approximately 11 to 8.5 and similarly its relatives show an increase of about 2 points in their expression values. This supports the assumption of the relatedness of these genes to *Id3 *in the learning process on Tomczak dataset. However, considering that *Id3 *is still very significant in Sartorelli, *Id3 *parent/children show no variation and simply are not significant. As a conclusion, this model failed to predict *Id3 *expression value and as a result the rank of *Id3 *dropped 132 places most probably due to the quality and biological variation of Sartorelli dataset. Since we aim to overcome the lack of overlap on the gene regulatory network studies across species and platforms, the natural extension of the work in this paper would be to explore how this model can be used on datasets from multiple biological systems with increasing complexity. Moreover, it would be valuable to consider methods such as model averaging [34] that has been shown better generalization in classifier's accuracy. Consequently, it improves the performance of classifiers in identifying the most informative genes and avoids deterioration of cases like *Id3*. Furthermore, dynamic Bayesian networks can be adopted when learning from time-series data in order to handle auto-regulation and feedback loops, two key components of regulatory networks in biological data [35,36].

## Conclusions

In this study, we have investigated a number of different Bayesian classifiers and datasets for identifying firstly, subsets of genes that are related to myogenesis and muscle differentiation, and secondly the use of cleaner and more informative datasets in modelling more biologically complex datasets. We have shown that an appropriate combination of simpler and more informative datasets produce very good results, whereas models learnt on genes selected from more complex datasets performed poorly. We concluded that simpler datasets can be used to model more complex ones and capture the interactions among genes. Moreover, we have described that highly predictive and consistent genes, from a pool of differentially expressed genes, across independent datasets are more likely to be fundamentally involved in the biological process under study. In three published datasets, we have demonstrated that these models can explain the myogenesis-related genes (genes of interest) significantly better than others (*P *< 0.004) since the improvement in their rankings is much more pronounced. These results imply that gene regulatory networks identified in simpler systems can be used to model more complex biological systems. In the example of muscle differentiation, a myogenesis-related gene network may be difficult to derive from in vivo experiments directly due to the presence of multiple cell types and inherently higher biological variation, but may become evident after initial training of the network on the cleaner in vitro experiments. In order to validate our approach, firstly, we evaluated our model on synthetic datasets and secondly we performed comparisons between our approach and the method of Lai *et al*. [32] which we call concordance model. It is shown that our model performs comparably in improving the ranks of informative genes and deteriorating the ranks of uninformative ones, but that the improvement of ranks for myogenesis-related genes is much more pronounced whilst additionally modelling the interactions among genes. However, it is necessary to develop other statistical measures so that the model can be quantified to distinguish different degrees of complexities and platforms whilst handling the auto-regulation and feedback loops within the network.

## Authors' contributions

SYA, PACH and AT contributed equally to methods development and drafting the paper. PACH provided the biological insight on the datasets related to muscle differentiation. AT designed the algorithms and SYA developed the codes. PACH and AT supervised the study. All authors analyzed the data, read and approved the final manuscript.

## Algorithm 1 - Multi Data Gene Identification Algorithm

Input: *{TrainD, TestD_1_,...TestD_M_, folds}*

   **for ***k *= 1:*folds*

      **Learn ***BN ***using Algorithm 2 on training folds of**

      *TrainD*

      **Score ***SSE ***on test fold ***k ***of ***TrainD*

      **Score ***SSE ***on all independent test datasets**

      {*TestD_1_...TestD_M_*}

   **end for**

   **Calculate variance of ***SSE ***over all ***k ***folds**

   **on ***TrainD ***and **{*TestD_1_...TestD_M_*}

   **Create gene rankings: ***trainR_SSE*, *train_var*,

   {*testR_SSE_1_*...*testR_SSE_M_*} **and**

   {*testR_var_1_*...*testR_var_M_*} **by ordering the genes**

   **on the respective ***SSE ***and ***variance ***scores**

**Output:**: *trainR_SSE, train_var*,

   *{testR_SSE_1_...testR_SSE_M_}*

   *{testR_var_1_...testR_var_M_}*

## Algorithm 2 - Simulated Annealing Structure Learning

Input: *t_0_, maxfc, D*

   *fc = 0, t = t_0_, t_n _= 0.001*

   *c = (t_n_/t_0_)^1/maxfc^*

   **Initial ***bn ***to a Bayesian classifier with no inter-gene links**

   *results = bn*

   *oldscore = score(bn)*

   **while ***fc < maxfc ***do**

      **for each operator do**

         **apply operator to ***bn*

         *newscore = score(bn)*

         *fc = fc + 1*

         *dscore = newscore-oldscore*

         **if ***newscore>oldscore ***then**

            *result = nbc*

         **else if ***r(0,1) < e^dscore/t ^***then**

            **Undo the operator**

         **end if**

      **end for**

      *t = t *× *c*

   **end while**

**Output: ***result*

## Supplementary Material

Additional file 1 This file contains 11 additional figures illustrating the results of our study in full details, as well as more information on the generation of synthetic datasets and the results of the Kolmogorov-Smirnov test. Click here for file

## References

[B1] Bockhorst J Craven M Page D Shavlik J Glasner J A Bayesian approach to operon prediction Bioinformatics 2003 19 1227 1235 10.1093/bioinformatics/btg147 12835266

[B2] Segal E Shapira M Regev A Pe'er D Botstein D Koller D Friedman N Module networks: identifying regulatory modules and their condition specific regulators from gene expression data Nature Genetics 2003 34 166 176 10.1038/ng1165 12740579

[B3] Friedman N Linial M Nachman I Pe'er D Using Bayesian networks to analyze expression data Proceeding of the 4th International Conference on Computational Molecular Biology 2000 127 135 10.1089/106652700750050961 11108481

[B4] Xu X Wang L Ding D Learning module networks from genome-wide location and expression data FEBS Letters 2004 587 297 304 10.1016/j.febslet.2004.11.019 15589836

[B5] Grossman D Domingos P Learning Bayesian network classifiers by maximizing conditional likelihood Proceedings of the 21st International Conference on Machine Learning 2004 69 46 54

[B6] Peña JM Björkegren J Tegnér Learning dynamic Bayesian network models via cross-validation Pattern Recognition Letters 2005 26 2295 2308 10.1016/j.patrec.2005.04.005

[B7] Pearl J Fusion, propagation, and structuring in belief networks Artificial Intelligence 1986 29 241 288 10.1016/0004-3702(86)90072-X

[B8] Buntine WL A guide to the literature on learning probabilistic networks from data IEEE Transactions on Knowledge and Data Engineering 1996 8 195 210 10.1109/69.494161

[B9] Heckerman D Jordan MI A tutorial on learning with Bayesian networks Learning in graphical models 1998 Dordrecht: Kluwer Academic Publishers 301

[B10] Friedman N Koller D Being Bayesian about network Structure. A Bayesian approach to structure discovery in Bayesian networks Machine Learning 2003 50 95 125 10.1023/A:1020249912095

[B11] Friedman N Geiger D Goldszmidt M Bayesian network classifiers Machine Learning 1997 29 131 163 10.1023/A:1007465528199

[B12] Fielding AH Introduction to classification Cluster and classification techniques for the Biosciences 2007 1 Cambridge: Cambridge University Press 86

[B13] Tobler JB Molla MN Nuwaysir EF Green RD Shavlik JW Evaluating machine learning approaches for aiding probe selection for gene-expression arrays Bioinformatics 2002 18 S164 S171 12169544 10.1093/bioinformatics/18.suppl_1.s164

[B14] Stone M Cross-validatory choice and assessment of statistical predictions (with discussion) Journal of the Royal Statistical Society B 1974 36 111 147

[B15] Kohavi R Wrapper for performance enhancement and oblivious decision graphs PhD thesis 1995 Stanford University, Computer Science Department

[B16] Bulcke T Van den Van Leemput K Naudts B Van Remortel P Ma H Verschoren A De Moor B Marchal K SynTReN: a generator of synthetic gene expression data for design and analysis of structure learning algorithms BMC Bioinformatics 2006 7 43 10.1186/1471-2105-7-43 16438721 PMC1373604

[B17] Zhang C Lu X Zhang X Significance of gene ranking for classification of microarray samples IEEE Transactions on Computational Biology and Bioinformatics 2006 3 312 320 10.1109/TCBB.2006.42 17048468

[B18] Su J Zhang H Full Bayesian network classifiers Proceedings of the 23rd International Conference on Machine Learning 2006 148 897 904 full_text

[B19] Chickering DM Heckerman D Meek C Large-sample learning of Bayesian networks is NP-Hard Machine Learning Research 2004 5 1287 1330

[B20] Schwarz G Estimating the dimension of a model The Annals of Statistics 1978 6 461 464 10.1214/aos/1176344136

[B21] Lam W Bacchus F Learning Bayesian belief networks (an approach based on the MDL principle) Computational Intelligence 1994 10 1 31 10.1111/j.1467-8640.1994.tb00166.x

[B22] Cao Y Kumar RM Penn BH Berkes CA Kooperberg C Boyer LA Young RA Tapscott SJ Global and gene-specific analyses show distinct roles of Myod and Myog at a common set of promoters The EMBO Journal 2006 25 502 511 10.1038/sj.emboj.7600958 16437161 PMC1383539

[B23] Iezzi S Di Padova M Serra C Caretti G Simone C Maklan E Minetti G Zhao P Hoffman EP Puri PL Sartorelli V Deacetylase inhibitors increase muscle cell size by promoting Myoblast recruitment and fusion through induction of Follistatin Developmental Cell 2004 6 673 684 10.1016/S1534-5807(04)00107-8 15130492

[B24] Tomczak KK Marinescu VD Ramoni MF Sanoudou D Montanaro F Han M Kunkel LM Kohane IS Beggs AH Expression profiling and identification of novel genes involved in myogenic differentiation The FASEB Journal 2004 18 403 405 14688207 10.1096/fj.03-0568fje

[B25] Irizarry RA Hobbs B Collin F Beazer-Barclay YD Antonellis KJ Scherf U Speed TP Exploration, normalization, and summaries of high density oligonucleotide array probe level data Biostatistics 2003 4 249 264 10.1093/biostatistics/4.2.249 12925520

[B26] Murphy KP The Bayes net toolbox for Matlab Computing Science and Statistics: Proceedings of the Interface 2001 33

[B27] Jelier R Schuemie MJ Veldhoven A Dorssers LC Jenster G Kors JA Anni 2.0: a multipurpose text-mining tool for the life sciences Genome Biology 2008 9 R96 10.1186/gb-2008-9-6-r96 18549479 PMC2481428

[B28] Haynes BC Brent MR Benchmarking regulatory network reconstruction with GRENDEL Bioinformatics 2009 25 801 807 10.1093/bioinformatics/btp068 19188190 PMC2732301

[B29] Ma H Kumar B Ditges U Gunzer F Buer J Zeng A An extended transcriptional regulatory network of Escherichia coli and analysis of its hierarchical structure and network motifs Nucleic Acids Res 2004 32 6643 6649 10.1093/nar/gkh1009 15604458 PMC545451

[B30] Miron M Woody OZ Marcil A Murie C Sladek R Nadon R A methodology for global validation of microarray experiments BMC Bioinformatics 2006 7 333 10.1186/1471-2105-7-333 16822306 PMC1539027

[B31] Rhodes DR Barrette TR Rubin MA Ghosh D Chinnaiyan AM Meta-Analysis of Microarrays: Interstudy validation of gene expression profiles reveals pathway dysregulation in prostate cancer Cancer Research 2002 62 4427 4433 12154050

[B32] Lai Y Eckenrode SE She J A statistical framework for integrating two microarray data sets in differential expression analysis BMC Bioinformatics 2009 10 S23 10.1186/1471-2105-10-S1-S23 19208123 PMC2648727

[B33] Mac Nally R Regression and model-building in conservation biology, biogeography and ecology: the distinction between - and reconciliation of - 'predictive' and 'explanatory' models Biodiversity and Conservation 2000 9 655 671 10.1023/A:1008985925162

[B34] Madigan D Raftery AE Model selection and accounting for model uncertainty in graphical models using Occam's window Journal of the American Statistical Association 1994 89 1535 1546 10.2307/2291017

[B35] Shen-Orr SS Milo R Mangan S Alon U Network motifs in the transcriptional regulation network of Escherichia coli Nature Genetics 2002 31 64 68 10.1038/ng881 11967538

[B36] Lee TI Rinaldi NJ Robert F Odom DT Bar-Joseph Z Gerber GK Hannett NM Harbison CT Thompson CM Simon I Zeitlinger J Jennings EG Murray HL Gordon DB Ren B Wyrick JJ Tagne JB Volkert TL Fraenkel E Gifford DK Young RA Transcriptional regulatory networks in Saccharomyces cereviciae Science 2002 298 799 804 10.1126/science.1075090 12399584

