# Supplementary material for: The identification of informative genes from multiple datasets with increasing complexity
Source: BMC Bioinformatics. 2010 Jan 15;11:32. doi: 10.1186/1471-2105-11-32 (PMC2822754; doi:10.1186/1471-2105-11-32)
Supplement: Additional file 1 — This file contains 11 additional figures illustrating the results of our study in full details, as well as more information on the generation of synthetic datasets and the results of the Kolmogorov-Smirnov test. [file 1471-2105-11-32-S1.PDF]

Supplementary Materials

The Identification of Informative Genes from Multiple Datasets with Increasing Complexity

S. Yahya Anvar[1,2,*], Peter A.C. ’t Hoen[2] and Allan Tucker[1]

[1]Center for Intelligent Data Analysis, School of Information Systems, Computing and Mathematics, Brunel University, Uxbridge, Middlesex, UB8 3PH, UK.

[2]Center for Human and Clinical Genetics, Leiden University Medical Center, P.O. Box 9600, 2300 RC, Leiden, The Netherlands.

Top 100 Genes Selected from Tomczak

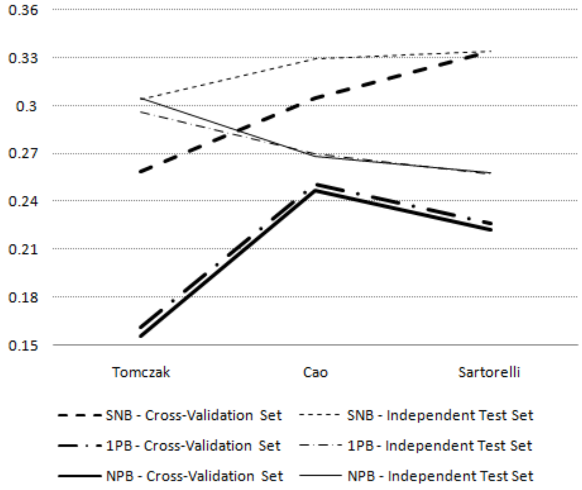

Fig. S1. The comparison of classifiers with increasing complexity. Three Bayesian network models (SNB, 1PB, and NPB) have been trained using cross-validation set and validated on independent datasets. An average error rate of the classifiers' prediction has been calculated for each gene (selected from Tomczak dataset) and an overall SSE on cross-validation set and independent test set are illustrated in this figure. These models have been trained on each dataset and validated on the other two datasets.

Top 100 and 50 Randomly Selected Genes from Tomczak

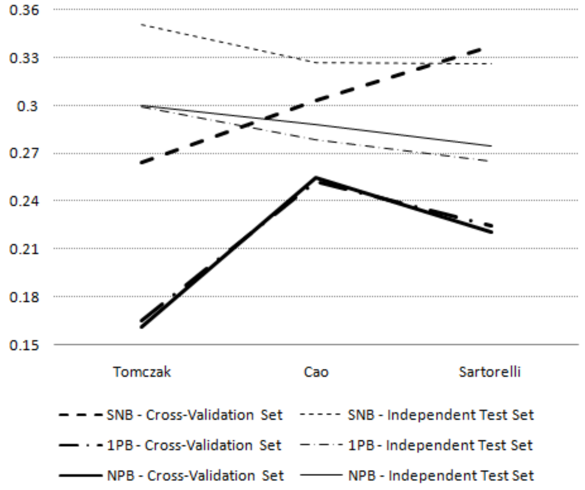

Fig. S2. The comparison of classifiers with increasing complexity. Three Bayesian network models (SNB, 1PB, and NPB) have been trained using cross-validation set and validated on independent datasets. An average error rate of the classifiers' prediction has been calculated for each gene (selected from Tomczak dataset) and an overall SSE on cross-validation set and independent test set are illustrated in this figure. These models have been trained on each dataset and validated on the other two datasets.

Top 100 Genes Selected from Cao

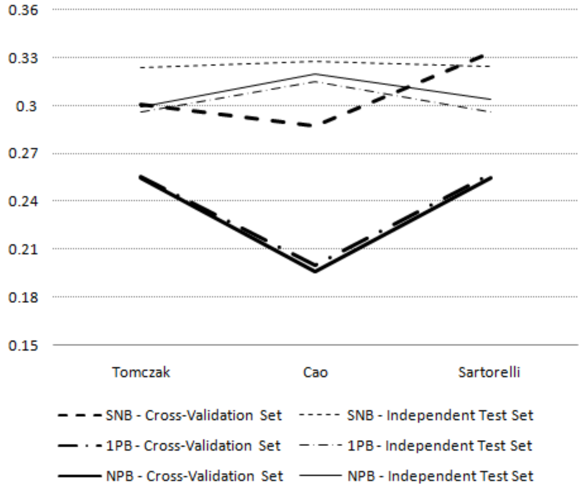

Fig. S3. The comparison of classifiers with increasing complexity. Three Bayesian network models (SNB, 1PB, and NPB) have been trained using cross-validation set and validated on independent datasets. An average error rate of the classifiers' prediction has been calculated for each gene (selected from Cao dataset) and an overall SSE on cross-validation set and independent test set are illustrated in this figure. These models have been trained on each dataset and validated on the other two datasets.

Top 100 and 50 Randomly Selected Genes from Cao

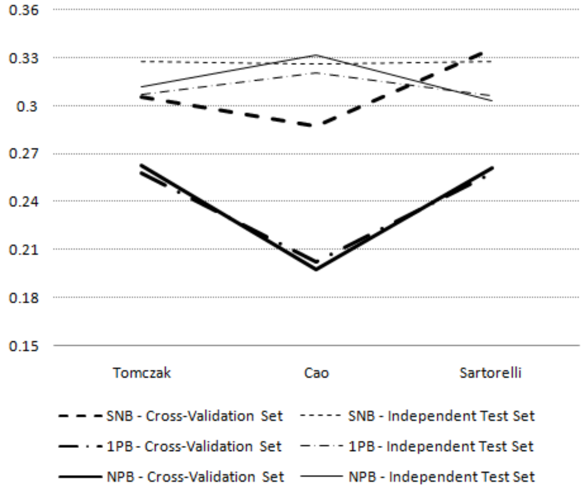

Fig. S4. The comparison of classifiers with increasing complexity. Three Bayesian network models (SNB, 1PB, and NPB) have been trained using cross-validation set and validated on independent datasets. An average error rate of the classifiers' prediction has been calculated for each gene (selected from Cao dataset) and an overall SSE on cross-validation set and independent test set are illustrated in this figure. These models have been trained on each dataset and validated on the other two datasets.

Top 100 Genes Selected from Sartorelli

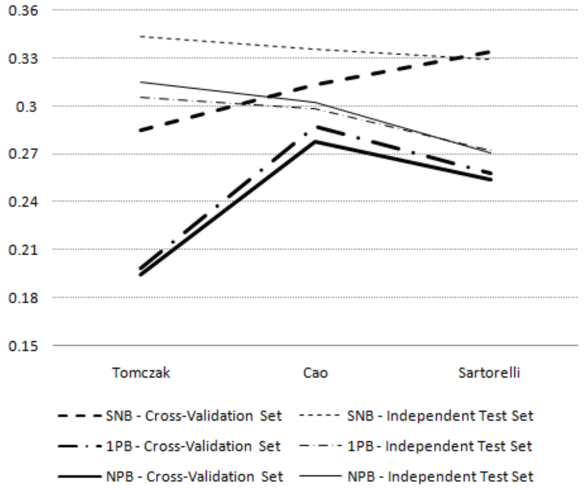

Fig. S5. The comparison of classifiers with increasing complexity. Three Bayesian network models (SNB, 1PB, and NPB) have been trained using cross-validation set and validated on independent datasets. An average error rate of the classifiers' prediction has been calculated for each gene (selected from Sartorelli dataset) and an overall SSE on cross-validation set and independent test set are illustrated in this figure. These models have been trained on each dataset and validated on the other two datasets.

Top 100 and 50 Randomly Selected Genes from Sartorelli

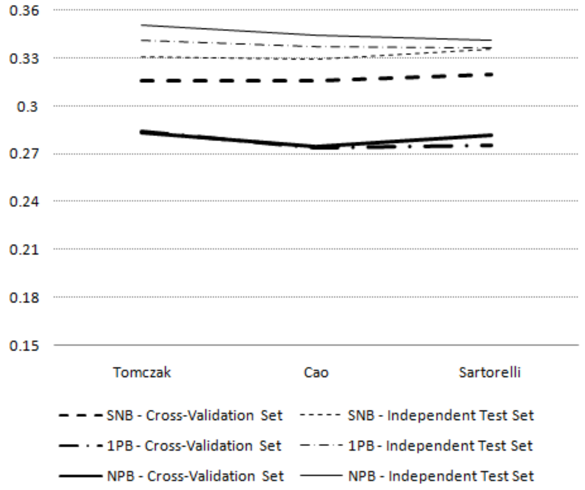

Fig. S6. The comparison of classifiers with increasing complexity. Three Bayesian network models (SNB, 1PB, and NPB) have been trained using cross-validation set and validated on independent datasets. An average error rate of the classifiers' prediction has been calculated for each gene (selected from Sartorelli dataset) and an overall SSE on cross-validation set and independent test set are illustrated in this figure. These models have been trained on each dataset and validated on the other two datasets.

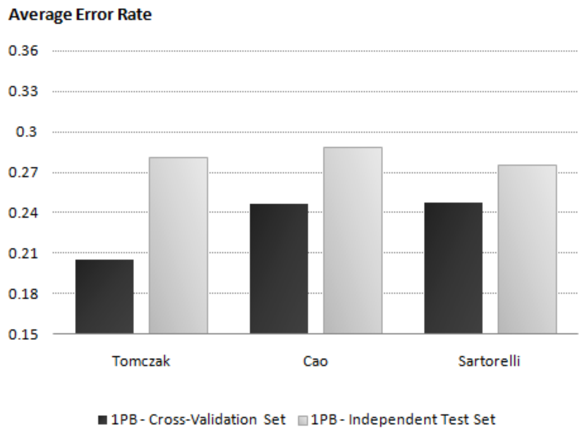

Fig. S7. The comparison of the differences between cross-validation set and independent test set on average error rates of 1PB classifier (extracted from figure 1).

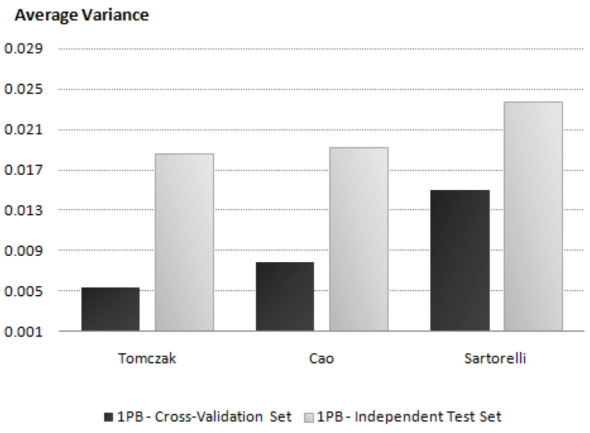

Fig. S8. The investigation of inference of adding more complexity to the model by adding 50 randomly selected genes as uninformative on 1PB classifier performance. In this figure we compare the average variance of 1PB classifier after adding 50 uninformative genes to the model.

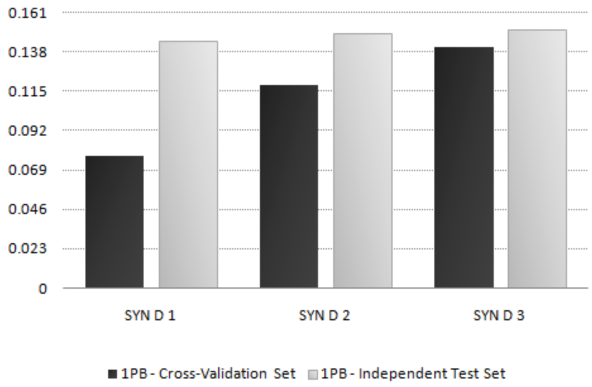

Fig. S9. This figure illustrates the performance of 1PB classifier on modeling three synthetic datasets generated using SynTReN application by manipulating the biological and experimental complexity. There is an increase of the biological variability on three datasets which matches an increase on the average error rate of models learnt.

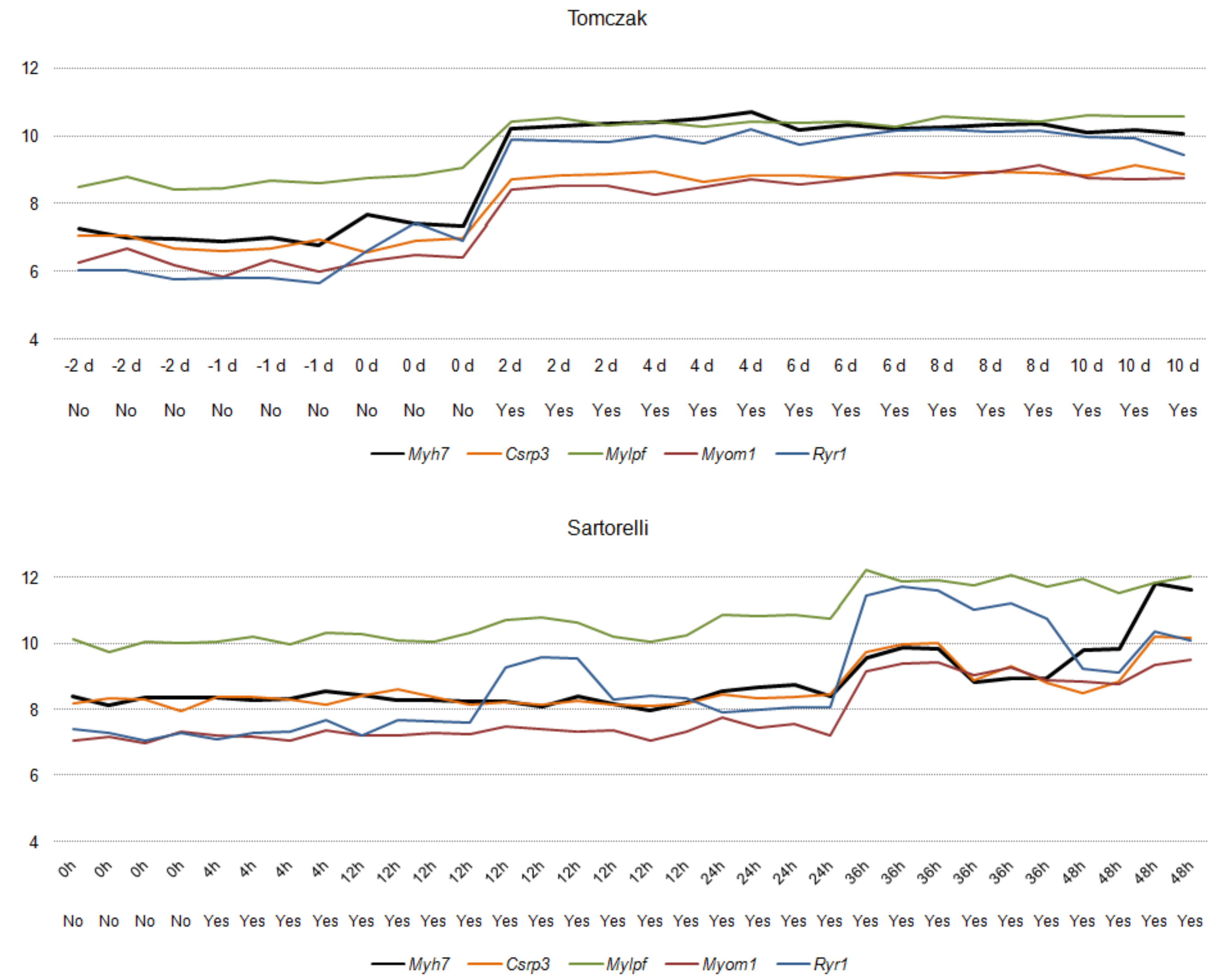

Fig. S10a. The expression level of *Myh7* along with its parent/children in both Tomczak and Sartorelli datasets. In Tomczak we can clearly see that there is a strong relationship between *Myh7* and the other 4 genes. Moreover, in Sartorelli dataset the correlation still exists between *Myh7* and *Csrp3*, *Mylpf*, *Myom1*, and *Ryr1* even though it is not as strong as Tomczak. This figure is an example of a large improvement of rank of a given gene after training on Tomczak. The x-axis represents both the time points and the differentiation status.

	Myh7	→	*Csrp3*	*Mylpf*	*Myom1*	*Ryr1*
Tomczak		Correlation	0.977551	0.980266	0.976016	0.988251
		P-Value	2.27E-16	5.56E-17	4.66E-16	1.92E-19
Sartorelli		Correlation	0.868451	0.763479	0.826257	0.59451
		P-Value	1.19E-10	3.73E-07	5.74E-09	0.000333

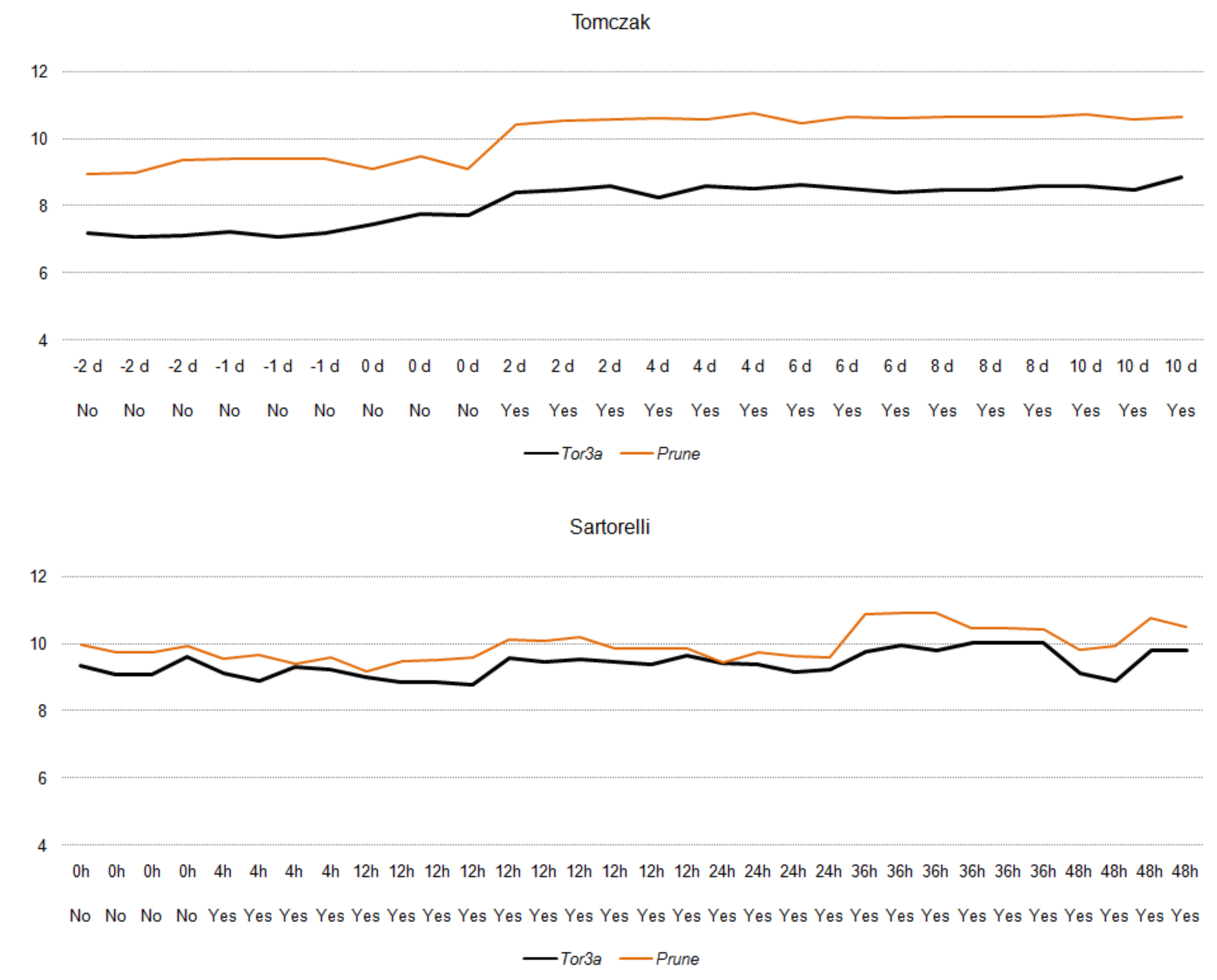

Fig. S10b. The expression level of *Tor3a* along with its parent in both Tomczak and Sartorelli datasets. In Tomczak we can clearly see that there is a good relationship between *Tor3a* and *Prune*. Moreover, in Sartorelli dataset the correlation still exists between *Tor3a* and *Prune*. This figure is an example of a large improvement of rank of a given gene after training on Tomczak. The x-axis represents both the time points and the differentiation status.

	Tor3a	⟶	*Prune*
Tomczak		Correlation	0.938426
		P-Value	1.25E-11
Sartorelli		Correlation	0.808676
		P-Value	2.15E-08

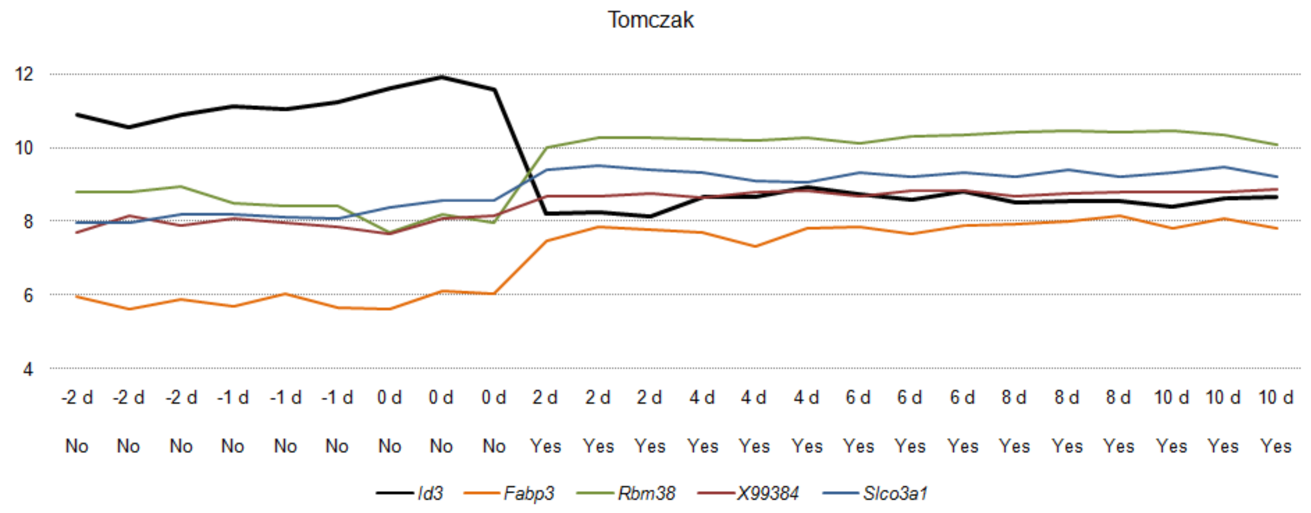

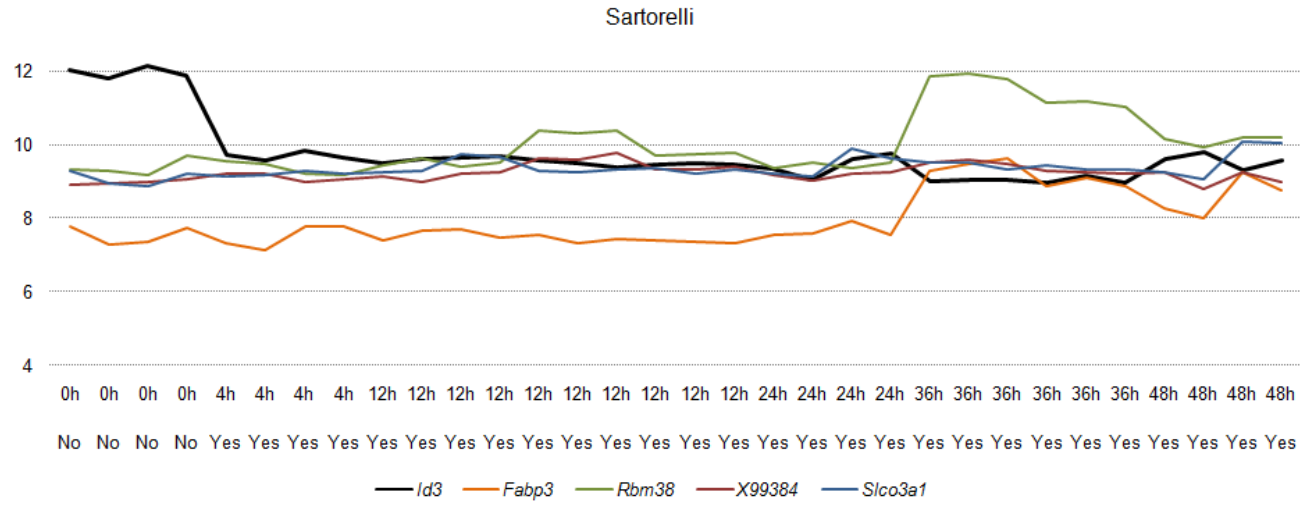

Fig. S11. The expression level of *Id3* along with its parent/children in both Tomczak and Sartorelli datasets. In Tomczak we can clearly see that there is an inverse relationship between *Id3* and the other 4 genes while Sartorelli dataset shows no significant correlations between *Id3* and *Fabp3*, *Rbm38*, *X99384*, and *Slco3a1*. This figure is an example of a large deterioration of rank of a given gene after training on Tomczak. The x-axis represents both the time points and the differentiation status.

Id3	→	*Fabp3*	*Rbm38*	*X99384*	*Slco3a1*
Tomczak	Correlation	-0.75923	-0.86705	-0.73964	-0.79244
	P-Value	4.72E-07	1.38E-10	1.32E-06	6.48E-08
Sartorelli	Correlation	-0.39787	-0.49415	-0.50536	-0.38395
	P-Value	0.024125	0.004046	0.003175	0.030047

Table S1: Differentiation Hypothesis. Investigating how well the models can separate the informative and uninformative genes from each other. Firstly, we ranked genes according to their average error rate and variance. Secondly, using Kolmogorov-Smirnov test and original ranking list, we explored which model can separate the informative genes from uninformative genes the best.

	Gene Selection		Error Rate (SSE)		Variance	
			Cross-Validation Set	Independent Test Set	Cross-Validation Set	Independent Test Set
»	Tomczak	Differentiation Hypothesis	TRUE	TRUE	TRUE	TRUE
		P-value	5.02E-24	9.77E-10	5.02E-24	3.68E-05
		Kolmogorov-Smirnov Test	0.880198	0.552871	0.880198	0.394257
		Average Performance (SSE/Variance)	**0.165259**	**0.298921**	**0.00537**	**0.018667**
	Cao	Differentiation Hypothesis	TRUE	TRUE	TRUE	TRUE
		P-value	1.89E-22	6.16E-06	1.91E-20	0.004314
		Kolmogorov-Smirnov Test	0.850297	0.425347	0.810693	0.295842
		Average Performance (SSE/Variance)	0.202472	0.320211	0.007819	0.019219
	Sartorelli	Differentiation Hypothesis	FALSE	TRUE	FALSE	FALSE
		P-value	0.443901	0.007507	0.527435	0.104457
		Kolmogorov-Smirnov Test	0.145941	0.282178	0.136832	0.205149
		Average Performance (SSE/Variance)	0.275287	0.336551	0.014939	0.023772

Table S2: The specification of three synthetic datasets generated for the purpose of the validation and reproduction of the result of applying our model on real microarray datasets used for this study. Three datasets have been generated on the well-described network structure of *E. coli* (Ma *et al.*, 2004) which contains 1330 number of nodes and 2724 interactions.

	SYN D 1	SYN D 2	SYN D 3
Burnin point	2000	2000	2000
Number of Experiments	15	15	15
Number of Samples per experiment	2	2	2
Number of Nodes	1000	1000	1000
Number of Background nodes	0	0	0
Probability for complex 2-regulator interactions	0.3	0.5	0.7
Biological noise	0.1	0.3	0.5
Experimental noise	0.1	0.3	0.5
Noise on correlated inputs	0.1	0.3	0.5
Number of External nodes	0	0	0
Number of Correlated external nodes	0	0	0
Sub network selection method	Cluster Addition		
Random seed	13	13	13
